# Factors Associated with Disclosure of Intimate Partner Violence among Women in Lagos, Nigeria

**DOI:** 10.5249/jivr.v1i1.15

**Published:** 2009-07

**Authors:** Leah Okenwa, Stephen Lawoko, Bjarne Jansson

**Affiliations:** ^*a*^Karolinska Institutet, Department of Public Health Sciences Stockholm, Division of Social Medicine, Sweden.

## Abstract

**Background:**

Though the prevalence of Intimate Partner Violence (IPV) remains high in less developed countries, data suggest that these figures may represent an underestimation considering that many women are unwilling to disclose abuse. This paper aims to determine women's willingness to report abuse, factors determining willingness to disclose IPV, and to whom such disclosure is made.

**Methods:**

A total of 911 women visiting reproductive health facility responded to the questionnaire, and the collected data was analyzed using multivariate analysis.

**Results:**

About 54% (n=443) of the participating women reported that would not disclose IPV. Among those willing to disclose abuse, 68% (n=221) would opt to disclose to close relatives in contrast to 32% (n=103) who would disclose to some form of institutions (i.e. religious leaders, law enforcement officers). Ethnicity, woman's own use of alcohol and autonomy in decision making such as having a say on household purchases, money use and visitation, independently predicted willingness to disclose IPV.

**conclusions:**

The role of family is still important in the Nigeria context and the implications for research and intervention are discussed.

## Introduction

Despite the inaction of laws and regulations against Intimate Partner Violence (IPV), the prevalence of IPV remains alarmingly high. Globally, the one-year prevalence ranges between 15 – 71%,^1,2^ with variations depending on factors such cultural norm, laws and other local conditions that favor/disfavor gender inequity. Yet, these figures may represent an under-estimation considering that significant proportions of women are unwilling to disclose abuse ^3,4^Disclosure of abuse is a vital step in the process of finding a lasting solution and breaking the abuse chain. Thus, unless victims are willing to disclose abuse and make use of available resources, screening for and eventual management of IPV may be heavily constrained.

Intimate Partner Violence is defined as a pattern of assaultive and coercive behaviors, including physical, sexual and psychological attacks, as well as economic coercion that adults or adolescents use against their intimate partners.^5^ Though women believe that screening gives victims support and information they need, they acknowledge that they have never disclosed disclose abuse in health care setting ^6,7,8^ The reasons for this discrepancy remain unclear but a likely explanation is that healthcare providers may lack adequate skills in promoting disclosure of abuse.^8^ In family planning and antenatal clinics three categories of women have been identified; women who will disclose abuse or fear of it; women who will not openly disclose abuse, but present with abuse-related physical symptoms (e.g. bruises) as well as reproductive health complications (e.g. lacerations and history of unexplained pregnancy complications); and finally women who live in an abusive relationship but do not report or show any signs associated with the abuse.^9^ These distinctions raise questions as to why some women disclose abuse while others do not.

A number of factors both at the organizational, household and individual level have been identified to explain why women may choose not to disclose abuse. Within the clinical setting constraints to disclosure reported by women interviewed, are the perceptions that clinicians lack interest in IPV and a lack of trust in the health care provider. This is further compounded by threats of more violence in retaliation from the partner and embarrassment.^10^ At the household level, women refrain from reporting abuse depending on the economic alternatives they have in the event of having to leave an abusive relationship. Lack of alternative economic resources may prevent women from reporting abuse.^11^ Moreover, potential for child abuse may hinder women from reporting abuse,^12,13^ suggesting that disclosure of abuse may fuel the intergenerational circle of violence from intimate partner violence to child abuse. At the individual level, there is evidence that women’s ability and willingness to disclose abuse is influenced by; her emotional strengths, her level of adherence to gender roles, decision-making autonomy, being asked about it, social empowerment and her perception of available social support^8,10,14^ It is however likely that these individual level factors are fueled by gender and social inequities at the societal level. Corroborating this argument, it is noted that factors such as ethnicity, culture, gender role definitions, kin and friendship networks may influence a woman’s perception of her options, the help she seeks, as well as the nature and scope of violence she experiences in an intimate relationship.^15^ Ethnicity and culture on their part have significant impact on women’s attitude to IPV such that an ethnic group that is more gender restrictive is more likely to condition women to agree or consent to wife beating.^16^ Normalization of IPV plays out significantly in Sub-Saharan African context. Recent data suggest that over 75% of the women believed that wife beating was justified when a woman does not leave up to her traditional normative roles (e.g. cooking and taking care of children).^2,17,18^

Societal, cultural and religious factors are not only important in determining whether women will report abuse or not, but also to whom such abuse will be reported. In many parts of Sub-Sahara Africa, marriage is considered a family and community affair rather than a private one. The role of the extended family therefore includes arbitrating in marital conflicts and finding ways to resolve them. Disclosure of abuse to some institutions such as law enforcement agencies is viewed as disrespect for the family. Indeed, authorities such as the police themselves condone such activity as women who dare to report are usually advised to go and settle with their husbands, denying women the opportunity to press charges and ultimately reducing their interest in seeking justice.^11,19,20^ Despite evidence that the major religions practiced in Nigeria i.e. Christianity, Islam and traditional religion all have teachings of female submission and obedience to the man as the head, findings reveal that some women are willing to disclose to religious leaders.^11^ However, distinctions between the categories of women who would make such reports are not yet clear.

Few studies coming from the African context have systematically examined the extent, nature and determinants of IPV disclosure. The objective of this study is: 1) to determine how willing women visiting an out-patient clinic in Lagos, Nigeria are to disclose abuse; 2) to whom such disclosure would be made; and 3) to determine factors which influence both willingness to disclose and the choice of to whom disclosure is made.

## Methods

**Study design and setting**

This cross-sectional study was conducted on woman attending out-patient clinic of the Obstetrics and Gynecology department of the Lagos University Teaching Hospital (LUTH), Nigeria. The hospital, as its name implies, is a teaching hospital affiliated with the Lagos University which is one of the oldest and largest institution in Nigeria. The hospital is a fee-paying federal government owned tertiary institution known for conducting quality research.

**Sampling procedure and participants**

A convenient sample of 934 women aged 15-49 years was obtained while they were visiting the Obstetrics and Gynecology clinic of LUTH. Women were included in the study if they were 15-49 years of age. The sample size needed for the study was established using a power analysis, assuming a binomial distribution. To obtain a statistical power over 90% which is considered as very good, a sample size of about 900 was necessary based on a statistical significance level of alpha=0.05, and an estimated average yearly probability of IPV occurring in developing countries of 0.125.^1^ Each participant responded to a questionnaire comprising of previously validated questions under the guidance of trained personal.

**Questionnaire**

A structured questionnaire covering demographic and health issues was administered verbally to the eligible women by trained field workers and voluntary participation emphasized. Study questionnaire was adapted from those commonly used by the World health organization and the demographic and health surveys and translated into the three major Nigerian languages, i.e. Hausa, Igbo and Yoruba. It was later back translated for validity. It covered women and their spouse’s background (such as education, use of alcohol), their reproductive history, utility of family planning methods, fertility preferences, child mortality, awareness of and precaution against sexually transmitted diseases, marriage and sexual behaviour, attitudes towards IPV, disclosure of IPV, psychosocial health outcomes, demographic, social and empowerment indicators as well as exposure to domestic violence. For the current paper, the questions of primary interest were those on attitudes towards IPV, exposure to IPV, demographic, social and empowerment indicators and willingness to disclose IPV.

## Measures

**Dependent variable**

Disclosure of IPV: participants responded to a hypothetical question posed as “would you disclose abuse?” The response options were “yes”, “no”, “don’t know”. Those responding “yes” to this question were probed further to find out to whom they would report. The response options to this follow-up question were: woman’s family only, husband’s family and close friends only, both families, religious leaders, the police and other types of social institutions. Subsequently, responses to this questions were dichotomized into two broader categories: 1) families and close friends (comprising those who would report to the woman’s family only, husband’s family and close friends only and those who would report to both families); and 2) the institutions (comprising of religious leaders, the police and other types of social institutions).

**Independent variables**

Attitudes to IPV was assessed using commonly used questions assessing IPV attitudes in the African context.^20,21,22^The questions assess whether participants would justify wife beating in five hypothetical situations: if the wife goes out with another man, neglects the children, argues with her partner, refuses to have sex with partner or cooks bad food/or food is served late. Answer options were “yes”, “no” or “don’t know”. An affirmative response to one or several of these questions was considered having a tolerant attitude towards IPV, while a “no” response on all five situations denoted a non tolerant attitude.

Exposure to IPV was assessed using a modified version of the Conflict Tactic Scale (CTS).^23^ Physical abuse was operationalized as being slapped, pushed, punched, choked, burnt on purpose, kicked and assaulted using knife or other weapons. Psychological abuse included being insulted, made to feel bad about self, belittled in front of other people, done things to scare or intimidate, and threatened to hurt respondent or someone she cares about. Sexual abuse included being physically forced to have sexual intercourse when she did not want to; having intercourse out of fear or forced to do sexually degrading or humiliating act. In this study, a victim of IPV was a woman who has experienced at least one of the forms of abuse described above.

Socio-demographic variables included: age; literacy (1=can read little or nothing, 2= can read whole sentences); religion (1=Catholic, 2=Protestant, 3=Muslim, 4=others); ethnicity (1=Yoruba, 2=Ibo, 3=others); earning income (1=yes, 2=no).

**Empowerment indicators included**

Access to information, assessed using frequency of reading newspaper, listening to radio, and watching TV all with response alternatives (1=almost everyday, 2=at least once weekly, 3=less than once weekly, 4=almost never/not at all); Decision autonomy, assessed by asking respondents whether they had a say on household expenditure, health care and household purchases with the following response options (1=complete say, 2=partial say, 3=no say). Women’s and households economic position, assessed by inquiring whether the woman contributes to household purchase, whether the household has problems making ends meet, or problems managing monthly expenditures with the following response options (1=yes, 2= no).

**Behavioural variables included**

respondent’s and partners use of alcohol and smoking habits (1=yes, 2=no) and polygamy.

**Ethical considerations**

National and local ethical clearance was granted by the Nigerian Institute of Medical Research, NIMR and the department of obstetrics and gynecology, LUTH before the questionnaire were administered. Ethical and safety recommendations set by the World Health Organization (WHO), which include training of and support to field workers, obtaining informed consent from participants, emphasis on voluntary participation as well as securing of anonymity were strictly followed.^25^ Moreover, the Institutional Review Board of the Nigeria Institute Medical Research approved the procedures, methodology and questionnaire content.

**Statistical analyses**

Data from the questionnaire were first entered into Microsoft excel and later transferred to SPSS program version 15.0, where analysis was done. Chi-square test was used to assess associations between willingness to disclose IPV and the independent variables. The significance level was set at p<0.05 for all statistical analysis. Logistics regressions analyses were used in the multivariable analysis to assess the independent contribution of the explanatory variables while adjusting for possible confounding. The direction and magnitude of associations were expressed as odds ratio. The significance level was set at p<0.05 for all statistical analysis.

## Results

**Willingness to disclose abuse and to whom**

The majority of women in the study (54%) were unwilling to disclose IPV (Table 1). Of those willing to report abuse, barely 1% indicated willingness to report to the police compared with over 28% willing to report to the man’s family, and 26% to religious leaders. In general, about 32% were willing to report to institutions contrasting with 68% willing to report to families and close friends (Table 1).”

**Table:** Table 1: **Frequency distribution of willingness to disclose and who disclosure is made to.**

	Frequency	Percentage
Willingness to disclose		
Yes	377	46.0
No	443	54.0
Disclosure Preference		
Husband’s family	93	28.7
Woman’s own family	46	14.2
Both families	63	19.4
Pastor /Imam	86	26.5
Husband’s friends	19	5.9
Police	3	0.9
other (specify)	14	4.3
Families and close friends	221	68.2
Institutions	103	31.7

**Socio-demographic and behavioral factors vs. willingness to disclose abuse and to whom**

As exhibited in Table 2, religion impacted significantly with willingness to disclose abuse with women of Catholic and “other” denomination most willing to disclose abuse (χ^2^(3) = 7.9; p<0.05). Willingness to report abuse was more common among women who used alcohol (χ^2^ (1)= 16.5; p<0.001) and those whose partners used alcohol (χ^2^(1)= 5.1; p<0.05) in contrast with their peers who did not or whose partners did not use alcohol (Table 2).

**Table:** Table 2: **Factors influencing disclosure: Demographic Factors vs. Disclosure**

Variable			Willingness to disclose				Families only				Institutions only			
			N	n	%	P- value	N	n	%	P- value	N	n	%	P- value
AGE						0.215				0.092				0.559
15-24			81	45	55.6		39	28	71.8		39	11	28.2	
25- 34yrs			520	240	46.2		207	131	63.3		207	66	31.9	
35 – 44yrs			198	84	42.4		71	40	56.3		71	23	32.4	
45 – 49yrs			16	6	37.5		5	1	20.0		5	3	60.0	
EDUCATION						0.572				0.134				0.098
Primary			36	14	38.9		12	8	66.7		12	4	33.3	
Secondary			170	75	44.1		60	44	73.3		60	12	20.0	
Post secondary			605	277	46.8		247	147	59.9		247	85	34.4	
LITERACY						0.336				0.015				0.049
Can’t read /reads parts of sentence			61	24	39.3		22	19	86.4		22	3	13.6	
Able to read whole sentence			682	312	45.7		264	159	60.2		264	90	34.1	
RELIGION						0.049				0.498				0.002
Catholic			269	139	51.7		121	81	66.9		121	25	20.7	
Protestant			346	142	41.0		117	69	59.0		117	47	40.2	
Muslim			76	33	43.4		30	20	66.7		30	7	23.3	
Others			118	59	50.0		52	30	57.7		52	22	42.3	
ETHNICITY						0.110				0.313				0.044
Yoruba			357	163	45.7		143	95	66.4		143	36	25.2	
Ibo			339	146	43.1		123	75	61.0		123	42	34.1	
Others			110	60	54.5		51	28	54.9		51	22	43.1	
ALCOHOL						0.000				0.287				0.372
Yes			117	74	63.2		63	43	68.3		63	17	27.0	
No			700	301	43.0		259	158	61.0		259	85	32.8	
HUSBAND’S ALCOHOL INTAKE						0.023				0.181				0.075
Yes			232	119	51.3		105	72	68.6		105	25	23.8	
No			542	230	42.4		199	121	60.8		199	67	33.7	

Regarding women’s preferences for disclosure, illiterate women were more willing to disclose to families in general (χ^2^(1) = 5.9; p<0.05), but least willing to report to institutions (χ^2^(1)= 3.9; p<0.05) (Table 2). Catholic and Muslim women were less willing to disclose IPV to institutions (χ^2^(3)= 14.4; p<0.01) than Protestant and women of “Other” denominations (table 2). Likewise, ethnic Yoruba women were less willing to disclose to the institutions than women of Ibo and “other” ethnic groups (χ^2^(2)= 6.2; p<0.05) (Table 2).

**Empowerment indicators vs. willingness to disclose abuse and to whom**

With regards to autonomy in decision concerning domestic life, women who had a say on household expenditure (χ^2^(2)=19.2; p<0.001), say on household purchase (χ^2^(2)=15.5; p<0.001), say on visiting family & friends (χ^2^(2)= 15.2; p<0.001); say on number of children to have and when to have children (χ^2^(2)= 8.2; p<0.05) were more willing to disclose abuse than their counterpart with “no say” in these respects (Table 3). Regarding women’s preferences for disclosure, women who “rarely” or “never” watch TV were less willing to disclose IPV to families (χ^2^(3)= 16.7; p<0.001) or to institutions (χ^2^(3)= 20.3; p<0.001) (Table 3).

**Table:** Table 3: **Factors influencing disclosure: Social empowerment vs. disclosure**

Variable			Willingness to disclose				Families only				Institutions only			
			N	n	%	P- value	N	n	%	P- value	N	n	%	P- value
READS NEWSPAPAPER						**0.647**				**0.699**				**0.377**
Almost everyday			239	117	49.0		106	67	63.2		106	33	31.1	
At least once weekly			308	134	43.5		111	65	58.6		111	41	36.9	
Less than once weekly			85	39	45.9		34	21	61.8		34	11	32.4	
Almost never/not at all			186	87	46.8		73	49	67.1		73	18	24.7	
LISTENS TO RADIO						0.179				0.776				0.602
Almost everyday			482	208	43.2		179	112	62.6		179	54	30.2	
At least once weekly			174	90	51.7		74	47	63.5		74	24	32.4	
Less than once weekly			61	32	52.5		28	15	53.6		28	12	42.9	
Almost never/not at all			103	47	45.6		43	28	65.1		43	13	30.2	
WATCHES TV						0.161				0.001				0.0001
Almost everyday			726	327	45.0		281	176	62.6		281	88	31.3	
At least once weekly			51	29	56.9		24	17	70.8		24	5	20.8	
Less than once weekly			26	11	42.3		10	1	10.0		10	9	90.0	
Almost never/not at all			10	7	70.0		7	7	100		7	0	0.0	
SAY ON MONEY USE						0.0001				0.182				0.147
Complete say			383	200	52.2		182	117	64.3		182	130	71.4	
Partial say			215	74	34.4		60	32	53.3		60	35	58.3	
No say			64	24	37.5		19	14	73.7		19	14	73.7	
SAY ON HEALTH CARE						0.332				0.981				0.243
Complete say			241	114	47.3		98	61	62.2		98	66	67.3	
Partial say			311	142	45.7		124	74	62.1		124	90	72.6	
No say			193	78	40.4		69	42	60.9		69	42	60.9	
SAY ON HOUSEHOLD						0.0001				0.318				0.509
Complete say			101	49	48.5		44	31	70.5		44	32	72.7	
Partial say			250	134	53.6		115	66	57.4		115	80	69.6	
No say			396	151	38.1		132	81	61.4		132	85	65.4	
SAY ON VISITING FAMILY & FRIENDS						0.0001				0.760				0.358
Complete say			167	82	49.1		75	47	62.7		75	54	72.0	
Partial say			440	212	48.2		179	112	62.6		179	123	68.7	
No say			139	42	30.2		39	22	56.4		39	23	59.0	
SAY ON NUMBER & WHEN TO HAVE CHILDREN						0.017				0.707				0.655
Complete say			48	27	56.3		25	17	68.0		25	18	72.0	
Partial say			546	252	46.2		222	135	60.8		222	151	68.0	
No say			99	33	33.3		28	16	57.1		28	17	60.7	

**Attitudes towards and exposure to IPV vs. willingness to disclosure abuse and to whom:**

Women who had ever experienced physical (χ^2^(1) = 9.3; p<0.01), psychological (χ^2^(1) = 3.7; p=0.052) and sexual IPV (χ^2^(1) = 11.7; p<0.01) were more willing to disclose abuse (Table 4). A similar trend was observed for experience of violence in the latest year. No association was found between having tolerant attitude to IPV and willingness to report abuse or to whom abuse would be reported (Table 4).

**Table:** Table 4: **Factors influencing disclosure: Attitudes and exposure to IPV vs. Exposure**

Variable			Willingness to disclose				Families only				Institutions only			
			N	n	%	P- value	N	n	%	P- value	N	n	%	P- value
Attitudes to IPV						0.228				0.822				0.197
Non-tolerant			506	241	47.6		207	130	62.8		207	71	34.4	
Tolerant			314	136	43.3		117	72	61.5		117	32	27.4	
Ever experienced physical IPV						0.002				0.913				0.550
No			643	278	43.2		237	149	62.9		237	76	32.1	
Yes			145	83	57.2		74	46	62.2		74	21	28.4	
Ever experienced psychological IPV						0.052				0.090				0.218
No			490	211	43.1		182	121	66.5		182	52	28.6	
Yes			297	149	50.2		128	73	57.0		128	45	35.2	
Ever experienced sexual IPV						0.001				0.223				0.102
No			662	285	43.1		246	150	61.0		246	82	33.3	
Yes			120	72	60.0		62	43	69.4		62	14	22.6	
Experienced physical IPV within the past year						0.007				0.809				0.884
No			748	333	44.5		285	177	62.1		285	91	31.9	
Yes			72	44	61.1		39	25	64.1		39	12	30.8	
Experienced psychological IPV within the past year						0.018				0.061				0.128
No			627	274	43.7		232	152	65.5		232	68	29.3	
Yes			193	103	53.4		92	50	54.3		92	35	38.0	
Experienced sexual IPV within past year						0.027				0.290				0.300
No			750	336	44.8		287	176	61.3		287	94	32.8	
Yes			70	41	58.6		37	26	70.3		37	9	24.3	
Ever experienced any type of IPV						0.006				0.953				0.780
No			410	169	41.2		146	91	62.3		146	47	32.2	
Yes			38	194	50.9		166	104	62.7		166	51	30.7	
Experienced any type of IPV within the past year						0.002				0.801				0.767
No			574	244	42.5		207	128	61.8		207	67	32.4	
Yes			246	133	54.1		117	74	63.2		117	36	30.8	

N=Number within category, n= number within category that is willing to disclose, % = N/n * 100 (i.e. proportion willing to disclose within category, p is the significance level for associations between independent variables and willingness to disclose

**Independent predictors of willingness to disclose IPV:**

As expressed by the odds ratios in Table 5, ethnicity, alcohol use and some measures of autonomy remained significantly associated with willingness to report IPV when adjusted for possible confounding variables in the logistic regression. Ibo ethnic group was less willing to report IPV than other ethnic groups. Women using alcohol, women who had say on household purchases, and say on visiting friends/relatives were more willing to disclose IPV than their peers who did not use alcohol and had no say on household purchases or visiting friends/relatives. All other variables did not reach statistical significance when possible confounding was adjusted for.

**Table:** Table 5: **Odds ratios indicating independent predictors of willingness to disclose IPV**

Independent variables	Adjusted a OR	(CI for OR)	P-value
Block 1	Willingness to disclose abuse		
AGE			
15-24	2.695	(0.500 –14.535)	0.249
25- 34yrs	3.108	(0.690 –13.995)	0.140
35 – 44yrs	2.156	(0.467 -9.946)	0.325
45 – 49yrs	1.00		
EDUCATION			
Primary	0.984	(0.326 –2.969)	0.978
Secondary	1.188	(0.667 –2.116)	0.558
Post secondary	1.00		
LITERACY			
Can read little /Nothing	1.096	(0.475 –2.531)	0.829
Able to read whole sentence	1.00		
RELIGION			
Catholic	1.103	(0.557 –2.187)	0.778
Protestant	0.832	(0.445 –1.554)	0.564
Muslim	0.760	(0.306 –1.889)	0.554
Others	1.00		
ETHNICITY			
Yoruba	0.814	(0.423 – 1.568)	0.538
Ibo	0.506	( 0.259 – 0.987)	0.046
Others	1.00		
ALCOHOL			
Yes	2.202	(1.123 – 4.318)	0.022
No	1.00		
HUSBAND’S ALCOHOL INTAKE			
Yes	0.959	(0.560 – 1.642)	0.880
No	1.00		
Block 2	Willingness to disclose abuse		
READS NEWSPAPAPER			
Almost everyday	1.168	(0.603 – 2.265)	0.645
At least once weekly	1.057	(0.574 – 1.948)	0.859
Less than once weekly	1.117	(0.505 – 2.471)	0.785
Almost never/not at all	1.00		0.967
LISTENS TO RADIO			
Almost everyday	0.543	(0.274 – 1.075)	0.080
At least once weekly	0.682	(0.324 – 1.439)	0.316
Less than once weekly	0.892	( 0.353 – 2.251)	0.809
Almost never/not at all	1.00		
WATCHES TV		
Almost everyday	0.580	(0.078 – 4.296)	0.594
At least once weekly	0.724	(0.082 – 6.366)	0.771
Less than once weekly	0.271	( 0.028 – 2.635)	0.261
Almost never/not at all	1.00		
SAY ON MONEY USE			
Complete say	0.986	(0.471 – 2.054)	0.970
Partial say	0.453	(0.204 – 1.008)	0.052
No say	1.00		
SAY ON HEALTH CARE			
Complete say	0.727	(0.391 – 1.351)	0.313
Partial say	0.607	(0.327 – 1.126)	0.114
No say	1.00		
SAY ON HOUSEHOLD PURCHASE			
Complete say	1.166	(0.598 – 2.273)	0.653
Partial say	1.858	(1.155 – 2.989)	0.011
No say	1.00		
SAY ON VISITING FAMILY & FRIENDS			
Complete say	2.581	(1.198 – 5.561)	0.015
Partial say	3.065	(1.491 – 6.300)	0.002
No say	1.00		
SAY ON NUMBER & WHEN TO HAVE CHILDREN			
Complete say	2.114	(0.781 – 5.721)	0.140
Partial say	1.386	(0.699 – 2.750)	0.350
No say	1.00		
Block 3	Willingness to disclose abuse		
Attitudes towards IPV			
Yes	1.414	(0.920 – 2.172)	0.114
No			
Physical IPV in past year		
Yes	1.095	(0.510 – 2.352)	0.817
No			
Psychological IPV past year			
Yes	0.701	(0.433 – 1.133)	0.147
No			

## Discussion

This study examined willingness to disclose IPV among women aged 15-49 years in Lagos, Nigeria and identified factors associated with such disclosure as well as preferences regarding to whom disclosure would be made. The results revealed that majority of the interviewed women (54%), would choose not to disclose IPV. This figure seems higher than those reported previously where between 37% and 42% choose not to disclose violence.^3,26^ These studies however addressed actual disclosure of abuse among abused women while our study addressed willingness to disclose abuse even among women never abused. Considering that willingness to disclose abuse may not directly translate to actual disclosure on the event of abuse, the higher figure observed in our study may have been expected. Among women willing to disclose abuse, almost twice as many opted for disclosure to close relatives (68%) in contrasted with disclosure to the institutions (37%), where only a modest 1% were willing to disclose to the police. These findings are in agreement with other research conducted within African context.^11,27,28^ These results further substantiate the role of the extended family in arbitrating marital conflicts, including violence, and suggest a divergence from capitalizing on established institutions purported to protect women from abuse. It is suggested that women perceive marital problems as their own^29^ thus constituting internal barriers. On the other hand, women refraining from disclosing IPV to the institutions could also be an indication that they lack trust in such institutions or that such institutions lack interest in domestic problems. Data from developed and other non-African context suggest that this may be the case.^3,30,31^ Further researches are warranted to investigate institutional readiness to assist abused women within African culture in Nigeria.

A number of the demographic variables were significantly associated with willingness to disclose abuse. Catholic women were most willing to disclose abuse when compared with other denominations, though they were, together with Muslim women, less willing to disclose to the institutions when compared with Protestants. Though these findings add to the literature suggesting that ethnicity and religion may affect women’s choices in terms of disclosure and acceptability of IPV,^14,15,32,33,34^ they may also be suggestive that institutional readiness to assist abused women may vary depending on their religious and ethnic affiliations. Further research is warranted to test the later hypothesis.

Our findings show that after the ethnic Igbo women, ethnic Yoruba women were more likely than women from “other” ethnic groups to disclose to families, (although this did not reach statistical significance). The reason for this might be that among the Yoruba, women enjoyed high status as mothers, sisters and daughters within the family. Like men, they hold leadership positions and authority within these matrilineages, but do not enjoy the same benefits as wives.^32,33^ It can thus be concluded that Yoruba women tend to report more to families bearing in mind their higher status as sisters and daughters.

Women having some form of autonomy in household decisions (i.e. say on expenditure, purchases, number of children to have and visiting friends) were more willing to disclose abuse than their peers lacking such autonomy. These results were confirmed in the multivariable analysis. It is suggested that women’s social and economic empowerment is likely to lessen her dependence on her partner.^35^ This independence is often reflected in her ability to speak out. Our findings are in line with other studies indicating the role of education in the empowerment of women to denounce intimate partner violence.^36,37,29^ Empowering factors such as education and access to information were also significant factors in our study regarding IPV disclosure. Women with little or no education preferred reporting to families and were less willing to disclose to institutions. A likely explanation is that education enlightens women on their options and thus empowering them to challenge traditional norms on gender inequality. Lack of access to information may also be another reason why women remain bound to tradition. Our findings seem to point in this direction as women without access to radio or television preferred to disclose to families more so than to institutions.

One of the factors influencing willingness to report IPV in our study is the experience of IPV in itself. Women who have experienced physical, psychological and sexual violence in general were more willing to report abuse when contrasted with non-abused peers, corroborating previous work where actual disclosure other than willingness to disclose have been studied.^29,38^ These findings could not however be confirmed in the regressions analysis suggesting a possible confounding effect warranting further investigation. Contrary to our expectations, women with tolerant attitudes towards IPV in our study did not differ from their peers with intolerant attitudes to IPV regarding willingness to disclose. This appears contradictory to theories linking exposure to intimate partner violence with tolerant attitudes towards violence itself among women.^39^ Capitalizing on these previous works, we had expected to observe higher willingness to disclose IPV among women with intolerant attitudes to IPV. Thus, the role of attitudes in disclosure of IPV deserves further investigation before firm conclusions can be drawn.

In practice, the implications for intervention/prevention program are enormous. The extended family remains a respected authority in resolving marital issues in the Nigerian culture. Prevention programs can capitalize on this by empowering the family unit by providing IPV related educational workshops, and improving their access to IPV prevention information, including information related to gender role issues. The importance of involving family in IPV prevention cannot be overemphasized. It is indeed suggested that lack of family support could be a barrier for victims of IPV, preventing them from taking steps towards ending their ordeal.^11^

Lack of willingness of women to disclose IPV to the institutions also has important implications for training of law enforcement as well as religious leaders to become more proactive in handling and dealing with reports of IPV. Studies also point to the important role of health providers in screening for IPV and suggest that women are more likely to disclose IPV if probed by their health care providers.^40,41^

To the best of our knowledge, this is the first time that data on underlying factors determining women choice to disclose IPV to family/relatives or various institutions is being presented. However, more research is warranted to validate this finding. There are few limitations to this study that should be noted. The cross sectional design does not allow for causal interpretation of the results. It is also important to note that willingness to disclose abuse does not directly translate to actual disclosure on the event of abuse. Caution is therefore warranted in interpretation of our findings. Furthermore, this study was conducted in one site using convenient sampling which limits the generalizability of the findings to other hospital settings or ethnic communities in Nigeria. Larger study is needed to assess determinants of IPV disclosure among women using a random sample that is representative of multiethnic, multicultural and multi-religious society like Nigeria. It is also important to add that though our study has identified a number of factors that may affect IPV disclosure, other prominent factors such as threat of increased violence in retaliation of a report have not been included in the analysis. Future research may need to incorporate such measures. Another limitation of the findings has to do with the lack of sample power to assess the independent role of spouse, family members, friends, and institutions, as a separate entity, in associations with IPV disclosures. Finally, the study sample was self-selected in that only women willing to participate were included until the required sample size was reached. Even though the interviewers reported that there were only a few women opting not to participate, the characteristics of these women remain unknown. Whether this non-response was systematic or not remains therefore unclear.
